# Multifunctional Analysis of CD4^+^ T-Cell Response as Immune-Based Model for Tuberculosis Detection

**DOI:** 10.1155/2015/217287

**Published:** 2015-08-03

**Authors:** Miriam Lichtner, Claudia Mascia, Ilaria Sauzullo, Fabio Mengoni, Serena Vita, Raffaella Marocco, Valeria Belvisi, Gianluca Russo, Vincenzo Vullo, Claudio M. Mastroianni

**Affiliations:** ^1^Department of Public Health and Infectious Diseases, Istituto Pasteur-Fondazione Cenci Bolognetti, Sapienza University, Piazzale Aldo Moro 5, 00185 Rome, Italy; ^2^Infectious Diseases Unit, Sapienza University, Corso della Repubblica 79, 04100 Latina, Italy

## Abstract

Mono- and multifunctional specific CD4^+^ and CD8^+^ T-cell responses were evaluated to improve the immune-based detection of active tuberculosis (TB) and latent infection (LTBI). We applied flow cytometry to investigate cytokines profile (IFN-*γ*, TNF-*α*, and IL-2) of T cells after stimulation with TB antigens in 28 TB-infected subjects (18 active TB and 10 LTBI) and 10 uninfected controls. Cytokines production by CD4^+^ T cells at single-cell levels was higher in TB-infected subjects than uninfected controls (P < 0.0001). Assigning to activated CD4^+^ T cells, producing any of the three cytokines, a cut-off >0.45%, it was possible to differentiate TB-infected (>0.45%) by uninfected subjects (<0.45%). Among TB-infected subjects, the frequencies of multifunctional CD4^+^ T cells, simultaneously producing all 3 cytokines, are lower in active TB than LTBI subjects (*P* = 0.003). Thus, assigning to triple-positive CD4^+^ T cells a cut-off <0.182%, TB-infected individuals could be classified as active TB subjects (<0.182%) or LTBI subjects (>0.182%). The magnitude of CD8^+^ T-cell responses showed no differences between active TB and LTBI. Multifunctional CD4^+^ T-cell responses could have the potential to identify at single time point subjects without TB infection and patients having active or latent TB.

## 1. Introduction

*Mycobacterium tuberculosis* (*Mtb*) infects more than 2 billion people worldwide; 90% of* Mtb*-infected individuals are able to resist overt tuberculosis (TB) disease determining the state of latency of infection (LTBI) [1]. Although latent and active TB disease are likely part of a dynamic spectrum [2, 3], individuals with LTBI are classically considered to be asymptomatic and not infectious; thus, the accurate classification of TB status is essential since treatment and prevention approaches are entirely different.

All existing tests for LTBI diagnosis, the tuberculin skin test (TST) and the newer interferon-gamma release assays (IGRAs), are acceptable but remain imperfect tests [4]. They represent indirect markers of* Mtb *exposure and provide immunological evidence of host sensitization to TB antigens. Both tests depend on cell-mediated immunity, and neither test can accurately differentiate between active TB and LTBI, distinguish reactivation from reinfection, or discriminate the various stages within the spectrum of* Mtb *infection [5, 6]. Thus, there is a need for newer biomarkers to classify patients at a single time point as having active TB, LTBI, or no infection.

Alternative immunological methods have been investigated in recent years [7–11]. In particular, multifunctional T cells, defined by their ability to coexpress two or more cytokines, have showed a better diagnostic yield than IGRA to detect TB infection [12–14] and have improved discrimination between active TB and LTBI [7–11, 15].

However, currently there is no consensus whether multifunctional T cells represent a marker of protective immunity or disease activity. Studies in animal models revealed a potential association of multifunctional Th1 cells with protective immunity to TB [16], but some recent studies in humans have implicated multifunctional Th1 cells in protective immunity against pulmonary disease [10, 17], while others have shown that these cells might merely reflect the presence of active disease [7, 9].

In the present study, we applied an intracellular cytokine flow cytometry (ICCFC) to investigate monofunctional and multifunctional* Mtb*-specific CD4^+^ and CD8^+^ T cells in active TB and LTBI adults. Based on our findings, we propose an immune-based approach, which could improve the identification at single time point of subjects with no TB infection or patients having active or latent TB.

## 2. Materials and Methods

### 2.1. Study Subjects

The study population included 38 subjects enrolled at the Department of Public Health and Infectious Diseases, “Sapienza” University, Rome, Italy. The subjects were firstly classified into 2 main groups: TB infected and uninfected subjects. TB infected people were subsequently classified as active TB and latent TB. In summary, 18 patients with active TB (age range, 26–61 years); 10 patients with LTBI (age range, 41–60 years); and 10 healthy subjects (age range, 27–43 years) were recruited.

Diagnosis of active TB was made on the basis of clinical and radiological findings and was confirmed by identification of* M. tuberculosis* with microbiological methods and/or histological examination of affected tissues. All patients presented tubercular lung involvement.

We classified as LTBI the subjects who tested positive for both TST and QFT-GIT. All subjects classified as LTBI had also one of the following risk factors: chest X-ray suggestive of prior TB infection (apical pleural thickening, pulmonary nodules, upper lobe bronchiectasis, interstitial granulomatous calcification, cavitation, and lymph node or pericardial calcification) and a history of exposure to a case of active TB, originating from an area with a high prevalence of TB infection. None of the individuals had clinical, radiologic, and microbiological evidence of active TB, and none had received prior TB treatment. The healthy subjects were unexposed individuals with no previous history of TB, no know TB contact and tested negative for TST and QFT-GIT.

Measurement of IFN-*γ* levels by IGRA and multifunctional analysis of CD4^+^ and CD8^+^ T cells were performed on the same blood samples collected from all patients.

The study received approval from the Local Ethics Committee (reference number 2669), and informed consent was provided by all subjects.

### 2.2. Tuberculin Skin Test and QuantiFERON TB Gold-In Tube (QFT-GIT)

After blood was drawn for the QFT-GIT assay, a TST (Biocine Test PPD, Chiron, Siena, Italy) was performed according to the Mantoux method by the same experienced operator, considering an induration of ≥10 mm as positive. The QFT-GIT assay (Cellestis Limited, Carnegie, Australia) was carried out and interpreted by the same trained technician, as per the manufacturer's instructions. Both operators were blind to the clinical status of the patients.

### 2.3. Intracellular Cytokine Flow Cytometry (ICCFC)

For intracellular cytokine flow cytometry, heparinized peripheral blood was collected, and 0.5 mL of whole blood was added to 3 test tubes containing, respectively, saline (negative control), phytohaemagglutinin (PHA), and TB antigens (ESAT-6, CFP-10, and TB 7.7) [18]. The test tubes were supplied with the QFT-GIT. The TB antigens are pools of overlapping peptides and are pooled together as a single stimulation condition.

Whole blood was costimulated with anti-CD28 plus anti-CD49d (5 *μ*L/mL, BD Bioscience, Pharmingen, Italy) as indicated by several authors [9, 10, 19], and Brefeldin A (10 *μ*g/mL) (Sigma-Aldrich) was immediately added to each tube as previously described [20]. In order to avoid aspecific stimulation related to CD28/CD49d costimulation, we added anti-CD28/CD49d antibodies to both negative (saline) and positive (PHA) control tubes and to TB antigens tubes (ESAT-6, CFP-10, and TB 7.7) and we further subtracted the background values (in terms of IL-2, IFN-*γ*, and TNF-*α*) from TB antigens tubes.

**Figure 1:**
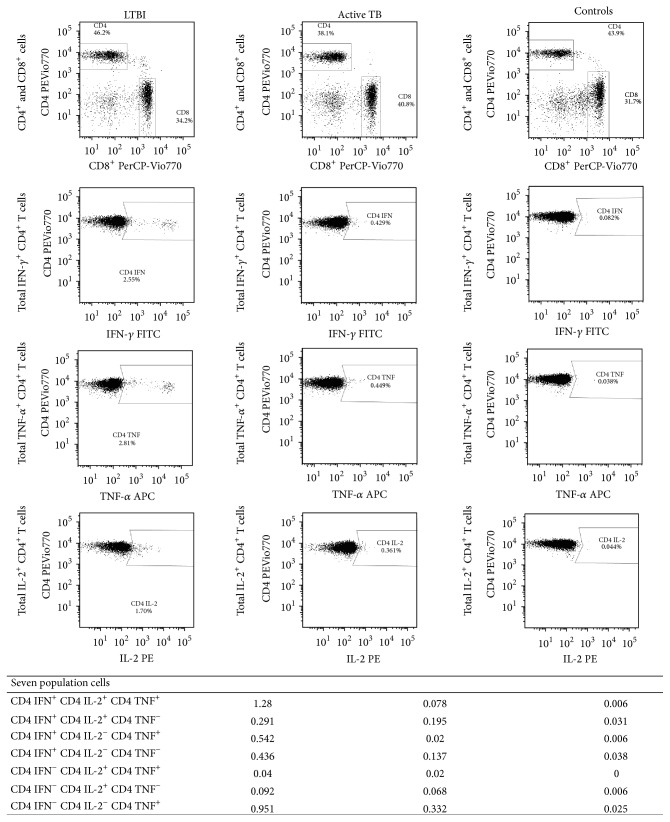
Representative flow cytometry “combination gates” analysis of CD4^+^ T cells of LTBI, active TB, and control subject under stimulation of TB antigens. Whole blood was analysed using a gating strategy to exclude debris and to identify CD4^+^ and CD8^+^ T cells on CD45^+^ lymphocytes. The subsequent analysis was on CD4^+^ gate to describe IFN-*γ*, IL-2, and TNF-*α* producing T cells. The percentages of the seven different population cells were showed at bottom and were defined in CD4^+^ cell gate on the basis of total IFN-*γ*, IL-2, and TNF-*α* producing cells.

In brief, after 18 hrs of incubation, the cell surface staining was performed with the following markers, anti-CD45-VioBlue, anti-CD4 PE-Vio770, and anti-CD8 PerCP (Miltenyi Biotec, Germany), and the red cells were lysed with 1 mL FACS lysing solution (BD Bioscience). Cells were then permeabilized with 0.5 mL FACS permeabilizing solution (BD Bioscience) and intracellularly stained with anti-IFN-*γ* FITC, anti-TNF-*α* APC, and anti-IL-2 PE (Miltenyi Biotec). Cells were fixed in 1% paraformaldehyde and analysed within 1 hr using a MACSQuant Analyzer flow cytometer (Miltenyi Biotec) after calibration and automatic compensation. We acquired at least 100,000 cells in the lymphocyte gate. FlowJo Software version 7.6.5 was used to perform a “combination gates” analysis. Seven different population cells were detected in CD4^+^ and in CD8^+^ cell gate on the basis of IFN-*γ*, IL-2, and TNF-*α* produced by CD4^+^ and CD8^+^ T cells (Figure 1). Background cytokine production in negative control (saline buffer) was subtracted from each stimulated condition. Intra-assay coefficient of variation and interassay coefficient variation were estimated and were <5% and <10%, respectively.

We classed T cells producing any of the 3 cytokines (IFN-*γ* or IL-2 or TNF-*α*) as “activated T cells,” those producing IFN-*γ* alone or in combination with IL-2 and/or TNF-*α* as “total IFN-*γ* ^+^ T cells,” those producing IL-2 alone or in combination with IFN-*γ* and/or TNF-*α* as “total IL-2^+^ T cells,” and those producing TNF-*α* alone or in combination with IL-2 and/or IFN-*γ* as “total TNF-*α* ^+^ T cells.” Similarly, we classed polyfunctional T cells (those simultaneously producing all 3 cytokines) as “IFN-*γ* ^+^ IL-2^+^ TNF-*α* ^+^ T cells.”

### 2.4. Statistical Analysis

GraphPad Prism Software version 5 (Software MacKiev) was used. Nonparametric Kruskal-Wallis ANOVA with Dunn's posttest comparison and nonparametric Mann-Whitney test was applied to compare T-cell frequencies and the percentage of cytokine-secreting cells between 3 or 2 groups of patients, respectively. Receiver operating characteristic (ROC) analysis was performed to calculate optimal cut-off values for both activated CD4^+^ T cells and polyfunctional CD4^+^ T cells. ROC curves were generated by plotting the sensitivity against 1 − specificity, and the area under the curve (AUC) with 95% confidence intervals (95% CIs) was calculated. All statistical analyses were two-sided and considered significant at *P* values < 0.05.

## 3. Results

### 3.1. TST and QFT-GIT Results

QFT-GIT was positive in 13/18 (72%), negative in 3/18 (17%), and indeterminate in 2/18 (11%) of active TB patients. As expected, QFT-GIF was positive in all 10 (100%) LTBI patients and in none of the healthy controls. The TST was positive in all LTBI and negative in all healthy controls, whereas it was positive in 11/18 (61%) and 7/18 (39%) of active TB patients.

### 3.2. Cytokine Flow Cytometry Analysis of* Mtb*-Specific CD4^+^ T Cells at the Single-Cell Level

**Figure 2:**
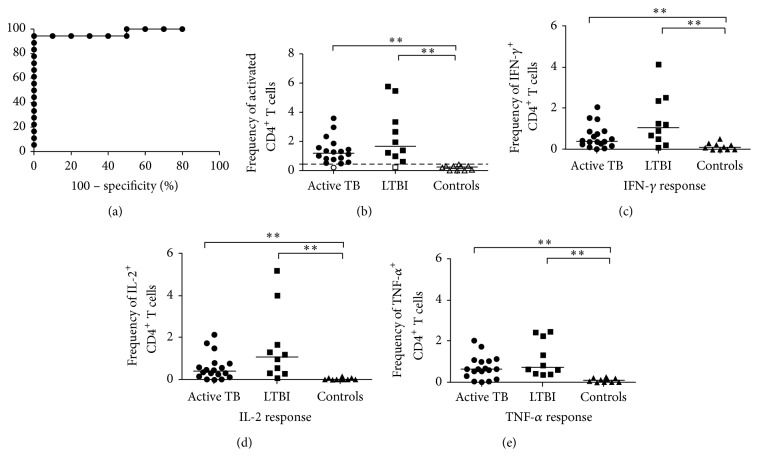
Analysis of cytokine production by CD4^+^ cells at the single-cell level. (a) ROC curve (plotting sensitivity versus 1 − specificity) to discriminate infected (active TB and LTBI) from uninfected patients. The area under curve (AUC) was 0.9722. (b) Analysis of activated* M. tuberculosis*-specific CD4^+^T cells producing any of the 3 cytokines (IFN-*γ*, IL-2, or TNF-*α*), using a cut-off to score responses as either positive or negative. The subjects were considered as positive (black) whether the frequency of CD4^+^ T cells was >0.45% and negative (white) when the frequency was <0.45%. Horizontal bars represent the median values and horizontal dashed line indicates the cut-off of 0.45%. ((c), (d), and (e)) Frequency of “total IFN-*γ* ^+^,” “total IL-2^+^,” and “total TNF-*α* ^+^”* Mtb*-specific CD4^+^T cells in active TB patients (*n* = 18), in LTBI patients (*n* = 10), and in healthy controls (*n* = 10) is shown. Horizontal bars represent the median values. Statistical analysis was performed using Kruskal-Wallis ANOVA with Dunn's posttest comparison and significant differences are indicated by asterisks (^**^ *P* < 0.01).

The expression of CD4^+^ T cells producing any of the 3 cytokines (IFN-*γ* or IL-2 or TNF-*α*) was assessed in patients with active TB, LTBI, and healthy controls, after simulation with* Mtb*-specific antigens. A significantly higher frequency of these CD4^+^ T cells was found in active TB patients (median 1.197%, range 0.219%–3.59%) and in LTBI patients (1.666%, 0.234–5.762%) if compared to healthy controls (0.246%, 0–0.423%; *P* < 0.0001 by Kruskal-Wallis test); on the other hand, no significant differences were found between the 2 infected group subjects. Following this observation, we performed a ROC analysis (Figure 2(a)) and a cut-off >0.45% for activated CD4^+^ T cells was found as the value allowing the best combination of sensitivity (94.44%, 95% CI: 72.2–99.8%) and specificity (100%, 95% CI: 69.15–100%; AUC 0.9722; 95% CI: 0.9141–1.030%, *P* < 0.0001) to differentiate* Mtb*-infected patients (active TB and LTBI) from healthy controls. Using this cut-off, we scored as positive 17/18 (95%) of active TB patients, 9/10 (90%) of LTBI patients, and none of 10 healthy controls (Figure 2(b)). Thus, the analysis of* Mtb*-specific CD4^+^ T cells allowed the discrimination between* Mtb*-infected and uninfected patients.

In another set of analyses, we compared the frequency of “total IFN-*γ* ^+^ CD4^+^ T cells,” “total IL-2^+^ CD4^+^ T cells,” and “total TNF-*α* ^+^ CD4^+^ T cells” (as defined in Section 2) in our 3 groups of the subjects. The frequencies of “total IFN-*γ* ^+^ CD4^+^ T cells” and “total IL-2^+^ CD4^+^ T cells” were higher in LTBI patients compared to those with active TB and healthy controls, although these differences attained statistical significance only between* Mtb-*infected (active TB and LTBI) and healthy subjects (*P* = 0.0014 for IFN-*γ*; *P* = 0.0001 for IL-2 by Kruskal-Wallis test) (Figures 2(c) and 2(d)). The frequency of “TNF-*α* ^+^ CD4^+^ T cells” was higher in patients with active and latent TB than in healthy controls (*P* = 0.0003 by Kruskal-Wallis test; Figure 2(e)).

Thus, the analysis of cytokine production by* Mtb*-specific CD4^+^ T cells at the single-cell level was unable to indicate TB status among the studied subjects not allowing a distinction between active TB and LTBI.

### 3.3. Multifunctional Cytokine Analysis of* Mtb*-Specific CD4^+^ T Cells

**Figure 3:**
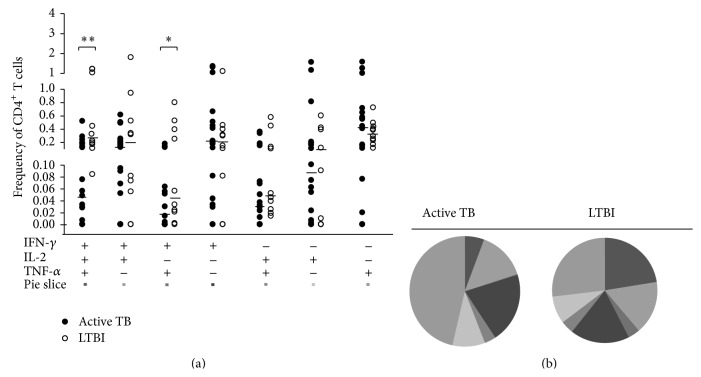
Multifunctional cytokine analysis of* M. tuberculosis*-specific CD4^+^ T cells. (a) Frequency of* Mtb*-specific CD4^+^ T cells producing all combinations of IFN-*γ*, IL-2, and TNF-*α* in active TB patients (*n* = 18, black circles) and in LTBI patients (*n* = 10, white circles). Horizontal bars represent the median values. Statistical analysis was performed using Mann-Whitney test and significant differences are indicated by asterisks (^**^ *P* < 0.01, ^*^ *P* < 0.05). (b) Pie charts represent the relative proportions of cytokine-producing T-cell subsets in each group after* Mtb*-specific stimulation. A key to colours used in the pie charts is shown at the bottom of the panel (a).

We analysed our samples for all possible combinations of intracellular expression of IFN-*γ*, IL-2, and TNF-*α* in cytokine-producing CD4^+^ T cells in subjects with active and latent TB. Significantly greater frequencies of both “IFN-*γ* ^+^ TNF-*α* ^+^” and “IFN-*γ* ^+^ IL-2^+^ TNF-*α* ^+^” CD4^+^ T-cell subsets were observed in LTBI patients compared to active TB patients (*P* = 0.003 and *P* = 0.034, respectively; by Mann-Whitney test) (Figure 3(a)). Conversely, the frequency of other single- or double-cytokine-secreting CD4^+^ T cells did not differ significantly between these two groups.

However, the cytokine profile (Figure 3(b)) revealed that in LTBI patients the proportion of polyfunctional CD4^+^ T cells producing the 3 cytokines simultaneously was greater (23%) compared to other double-cytokine-producing CD4^+^ T cells (IFN-*γ* ^+^ TNF-*α* ^+^ = 4%; IL-2^+^ TNF-*α* ^+^ = 4%; IFN-*γ* ^+^ IL-2^+^ = 16%), but similar to single-cytokine-producing CD4^+^ T cells (TNF-*α* ^+^ = 27%, IFN-*γ* ^+^ = 18%). In contrast, subjects with active TB showed a smaller proportion of polyfunctional CD4^+^ T cells (8%) and a predominance of CD4^+^ T-cell subset secreting TNF-*α* alone which constituted 46% of the total cytokine response.

**Figure 4:**
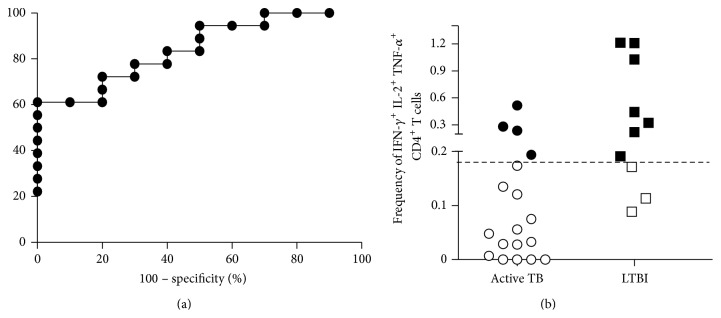
Differentiation between active and latent* Mtb*-infected subjects. (a) ROC curve (plotting sensitivity versus 1 − specificity) to discriminate active TB from LTBI patients. The area under curve (AUC) was 0.8444. (b) Analysis of triple-positive IFN-*γ* ^+^ IL-2^+^ TNF-*α* ^+^ CD4^+^ T cells, using a cut-off to score responses as either positive or negative. The subjects were considered as positive (black) whether the frequency of CD4^+^ T cells was >0.182% and negative (white) when the frequency was <0.182%. Horizontal bars represent the median values and horizontal dashed line indicates the cut-off of 0.182%.

Based on these differences, we performed a ROC analysis (Figure 4(a)) and cut-off < 0.182% for polyfunctional CD4^+^ T cells allowed the best combination of sensitivity (77.78%, 95% CI: 52.36–93.59) and specificity (70%, 95% CI: 34.75–93.33%; AUC 0.8444; 95% CI: 0.7021–0.9868%, *P* = 0.0002) to differentiate between active TB and LTBI subjects. Using this cut-off to score ICCFC responses as either positive or negative, we observed a positive response (>0.182%) in 4 out of 18 (22%) active TB patients and in 7 out of 10 (70%) LTBI patients (Figure 4(b)). In our hands, the frequency of polyfunctional CD4^+^ T cells which simultaneously produced IFN-*γ*, IL-2, and TNF-*α* may be indicative of LTBI status.

### 3.4. Specific CD8^+^ T-Cell Responses to* Mtb *Antigens in Intracellular Cytokine Flow Cytometry

**Figure 5:**
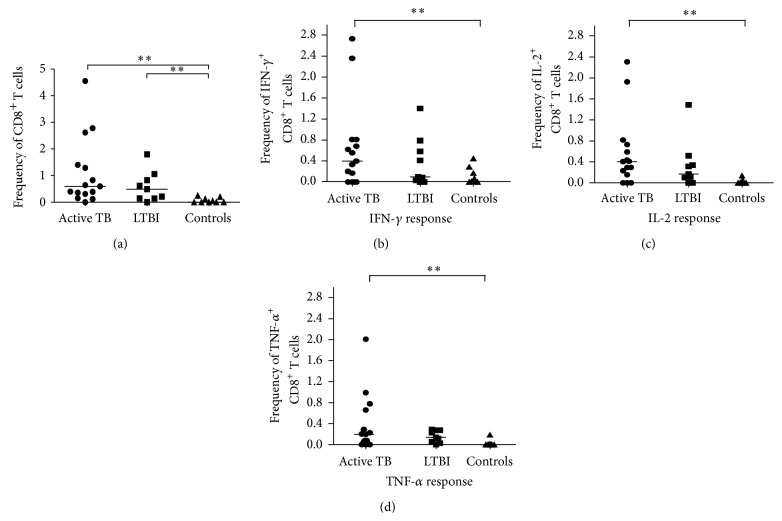
Analysis of cytokine production by CD8^+^ T cells at the single-cell level. (a) Frequencies of activated* Mtb*-specific CD8^+^T cells producing any of the 3 cytokines (IFN-*γ*, IL-2, or TNF-*α*) cells in active TB patients (*n* = 18), in LTBI patients (*n* = 10), and in healthy controls (*n* = 10) are show. ((b), (c), and (d)) Frequency of “total IFN-*γ* ^+^,” “total IL-2^+^,” and “total TNF-*α* ^+^”* Mtb*-specific CD8^+^T in active TB patients (*n* = 18), in LTBI patients (*n* = 10), and in healthy controls (*n* = 10) is shown. Horizontal bars represent the median values. Statistical analysis was performed using Kruskal-Wallis ANOVA with Dunn's posttest comparison and significant differences are indicated by asterisks (^**^ *P* < 0.01).

The analysis of activated CD8^+^ T cells, producing any of the 3 cytokines (IFN-*γ* or IL-2 or TNF-*α*) showed similar results to CD4^+^ T cells, revealing a significant greater frequency of activated CD8^+^ T cells in both active TB (median 0.599%, range 0–4.55%) and LTBI patients (0.489%, 0–1.796%) compared to healthy controls (0%, range 0–0.249%, *P* < 0.0020 by Kruskal-Wallis test), but no difference was found between two groups of infected individuals (Figure 5(a)). Likewise, the frequency of “total IFN-*γ* ^+^ CD8^+^ T cells” (Figure 5(b)), “total IL-2^+^ CD8^+^ T cells” (Figure 5(c)), and “total TNF-*α* ^+^ CD8^+^ T cells” (Figure 5(d)) was significantly higher in active TB patients compared to the other 2 groups of patients. These differences were only statistically significant between active TB subjects and healthy controls (*P* = 0.0045 for IFN-*γ* ^+^; *P* = 0.0033 for IL-2; *P* = 0.0078 for TNF-*α* by Kruskal-Wallis test).

**Figure 6:**
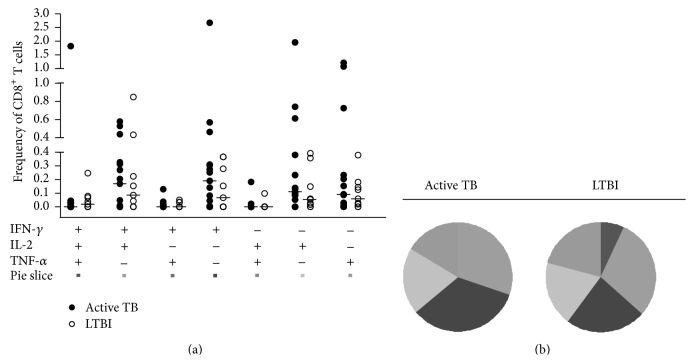
Multifunctional cytokine analysis of* Mtb*-specific CD8^+^ T cells. (a) Frequency of* Mtb*-specific CD8^+^ T cells producing all combinations of IFN-*γ*, IL-2, and TNF-*α* in active TB patients (*n* = 18, black circles) and in LTBI patients (*n* = 10, white circles). Horizontal bars represent the median values. Statistical analysis was performed using Mann-Whitney test. (b) Pie charts represent the relative proportions of cytokine-producing T-cell subsets in each group after* Mtb*-specific stimulation. A key to colours used in the pie charts is shown at the bottom of the panel (a).

As regards the cytokine profiles of* Mtb*-specific CD8^+^ T cells, we found no significant differences between active TB and LTBI subjects (*P* > 0.05 for each CD8^+^ T-cell subset by Mann-Whitney test; Figure 6(a)). Still, observing the pie charts (Figure 6(b)) the proportions of single-, double-, or triple-cytokine-secreting CD8^+^ T-cell subsets were comparable between two groups of individuals.

Hence, the evaluation of the* Mtb*-specific CD8^+^ T-cell responses in both monofunctional and polyfunctional analyses did not allow the distinction between active TB and LTBI subjects.

## 4. Discussion

In recent years, immunological response to* Mtb *has been extensively studied with the purpose of not only better understanding the TB pathogenesis but also improving diagnosis. The more recent IGRA offer some improvements over the TST, showing an excellent specificity for LTBI diagnosis and correlating well with the magnitude of exposure to* M. tuberculosis* [21]. Nevertheless, they have several known limitations including the reduced accuracy in immunocompromised subjects [22–24], the presence of conversions and reversions of results when serially applied in the same individuals [25–27], the inability to distinguish reactivation from reinfection, and the inability to accurately differentiate between LTBI and active TB [5, 6].

In the present study, using IGRA assay as a tool for TB detection, no significant differences in the average IFN-*γ* responses were observed among our TB and LTBI subjects, since similar percentages of positive IFN-*γ* responses were present in both of the 2 groups.

With a view to improving discrimination between active TB and LTBI, we applied an intracellular cytokine flow cytometry (ICCFC) to investigate monofunctional and multifunctional* Mtb*-specific CD4^+^ and CD8^+^ T cells.

Multifunctional T cells simultaneously secreting IFN-*γ*, TNF-*α*, and IL-2 play a critical role in the control of chronic bacterial and viral infections [28]. Changes in cytokine profiles could be a general feature of* Mtb*-specific T cells in TB infection, but limited and controversial data have made it difficult to define the role of these cells in TB [29]. Some studies support the concept that a higher proportion of triple-positive CD4^+^ T cells correlates with LTBI when comparing active TB and latent subjects, suggesting that this T-cell subset may be a surrogate marker of* Mtb* load and consequently of active replication control in LTBI subjects [10, 15, 17]. In contrast, others indicate a higher proportion of triple-positive CD4^+^ T cells in active TB than in LTBI subjects [7, 9], but the methodology used was different and so was the definition of LTBI.

The present study provides a detailed analysis of the frequency of cytokine-producing CD4^+^ and CD8^+^ T cells in* Mtb*-infected (active TB and LTBI) and uninfected subjects.

Regarding the CD4^+^ T-cell compartment, the first finding was that analysis of activated cells producing any of the 3 cytokines (IFN-*γ* or IL-2 or TNF-*α*) may help to differentiate* Mtb*-infected (active TB and LTBI) and uninfected subjects. In fact with a cut-off > 0.45% for activated CD4^+^ T cells, we scored as positive the 95% of active TB patients, the 90% of LTBI patients, and none of healthy controls, clearly differentiating* Mtb*-infected patients from healthy controls. It is interesting to note that active TB patients showed a low response using TST and QFT (only 61% and 72%, resp.) confirming that routinely immunological tests were not useful in diagnosis of active disease. Our data suggest that analysis of activated CD4^+^ T cells has a good sensitivity in active TB.

Also the analysis of CD4^+^ T cells secreting IFN-*γ* alone, IL-2 alone, and TNF-*α* alone, revealed a prevalent frequency of all these T cells in infected compared to uninfected subjects. Still, it is interesting to note a slightly higher frequency of both “all IFN-*γ* ^+^” and “all IL-2^+^” CD4^+^ T cells in LTBI patients with respect to active TB patients, in line with recent studies showing that CD4^+^ T cells producing IFN-*γ* and IL-2 have been implicated in protective immunity to TB [30, 31]. Taken together, monofunctional analysis was able to distinguish infected and uninfected subjects but was unable to indicate TB status not allowing a clear distinction between active and latent infection.

Moreover searching for a difference between active and latent TB, we performed a broad characterization of the functional profiles of* Mtb*-specific CD4^+^ T cells to determine all combinations of intracellular expression of IFN-*γ*, IL-2, and TNF-*α*. These findings revealed that latent infection is associated with an increased frequency of two CD4^+^ T-cell subsets, those producing IFN-*γ*, IL-2, and TNF-*α* simultaneously (triple-positive) and those producing IFN-*γ* in combination with TNF-*α*, indicating a possible protective role of these cells population in maintaining latent inflection. Indeed, we found that multifunctional CD4^+^ T cells, in particular those producing 3 cytokines simultaneously, provided the best discrimination between active and latent infection. In fact using a cut-off of 0.182% for triple-positive CD4^+^ T cells, most LTBI subjects (70%) showed a positive response, whereas the majority of active TB patients (78%) have a response below the cut-off.

Taken together, the detection of less than 0.182% of triple-positive CD4^+^ T cells is strongly indicative of active TB with a specificity of 70%, whereas frequencies of these cells above 0.182% could be indicative of LTBI with sensitivity of 77.7%.

Most notably and in contrast with latent infection, our subjects with active TB disease had a predominance of CD4^+^ T cells secreting TNF alone which constituted 46% of the total cytokine response. This is in agreement with previous studies showing that TNF production is a major component of the immune response to* Mtb*-antigen in active disease and thus the most reliable marker for diagnosing TB disease [7, 10, 11, 32, 33]. TNF expansion could be responsible for a high degree of inflammation, which could be linked to tissue damage and lung lesions rather than protection and control of the pathogen.

According to what has been assumed to be the hallmark of a protective CD4^+^ T-cell response in various models of human viral infections [28, 34, 35], we found a significantly higher proportion of multifunctional IFN-*γ* ^+^ IL-2^+^ TNF-*α* ^+^ CD4^+^ T cells in subjects with LTBI, which are able to control* Mtb *replication compared with those with current TB disease, in which CD4^+^ T cells secreting TNF alone dominated the* Mtb*-specific response.

As regards the CD8^+^ T-cell compartment, Rozot et al. [36, 37] recently indicated that* Mtb*-specific CD8^+^ T-cell responses can be detected predominantly in patients with active TB as compared to LTBI subjects, suggesting a correlation between CD8^+^ T-cell responses and high antigen burden [19, 38]. This hypothesis is supported by a recent study performed in children showing that* Mtb*-specific CD8^+^ T cells were detected in active TB disease but not in healthy children recently exposed to* Mtb*, despite the fact that similar frequencies of CD4^+^ T cells were present in both groups [39].

Moreover, little is known about the size, quality, and specificity of* Mtb*-specific CD8^+^ T-cell responses during active and latent infection. In our study, the monofunctional analysis allowed a distinction between infected and uninfected subjects, following the same trend as in CD4^+^ T cells. Conversely, the polyfunctional analysis of CD8^+^ T-cell responses showed no significant differences between active and latent infected patients.

**Figure 7:**
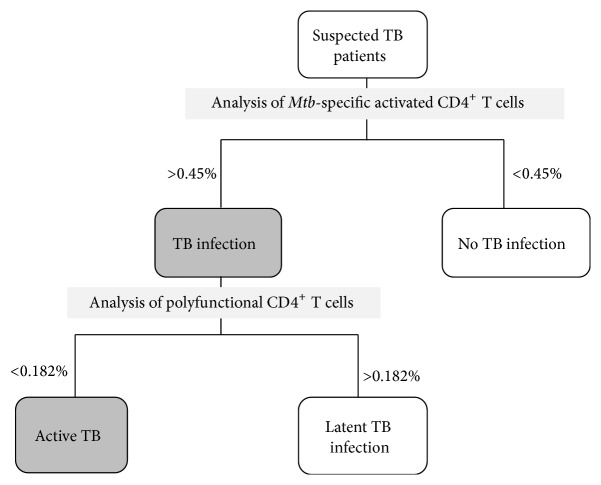
Proposed immune-based model for detection of active and latent TB. Taking into account the analysis of activated CD4^+^ T cells, it is possible to differentiate* Mtb*-infected subjects (>0.45%) by uninfected subjects (<0.45%). With the analysis of multifunctional CD4^+^ T cells, simultaneously producing all 3 cytokines, infected individuals may be classified as active TB subjects (<0.182%) or as LTBI subjects (>0.182%).

The present study has some limitations, such as the relatively small number of patients within each clinical group and the lack of a prospective analysis. Nevertheless, the ICCFC assessment of multifunctional* Mob*-specific CD4^+^ T cells enabled us to determine the different clinical stage of TB infection. In this respect we propose an immune model (Figure 7) which, with a cut-off of 0.45% for activated CD4^+^ T cells, may initially discriminate* Mtb*-infected (active TB and LTBI) patients (>0.45%) from uninfected subjects (<0.45%) with a specificity of 100%. Then, the infected individuals may be classified as active TB subjects if they showed frequencies of triple-positive T cells less than 0.182% and as LTBI subjects if the frequencies of triple-positive T cells are instead above 0.182% with a sensitivity of 77.78% and a specificity of 70%.

## 5. Conclusion

Multifunctional flow cytometry analysis of specific CD4^+^ T-cell response may represent a simple and rapid immune-based approach to distinguish between* Mtb*-infected and uninfected subjects. The more interesting result of the study is the increased number of active TB patients detected with multifunctional analysis of CD4^+^ T-cell response in comparison to QTF-GIT or TST. As general use as a clinical diagnostic test in order to identify patients with active versus latent TB infection, this immunological approach needs to be validated in a larger and prospective study and to be extended to other forms of active tuberculosis.
